# Polymyalgia rheumatica and giant cell arteritis—three challenges—consequences of the vasculitis process, osteoporosis, and malignancy: A prospective cohort study protocol

**DOI:** 10.1097/MD.0000000000007297

**Published:** 2017-06-30

**Authors:** Amir Emamifar, Søren Hess, Oke Gerke, Anne Pernille Hermann, Helle Laustrup, Per Syrak Hansen, Peter Thye-Rønn, Niels Marcussen, Frank Svendstrup, Rannveig Gildberg-Mortensen, Jacob Christian Bang, Ziba Ahangarani Farahani, Stavros Chrysidis, Pia Toftegaard, Rikke Asmussen Andreasen, Sebastian le Greves, Hanne Randi Andersen, Rudolf Nezlo Olsen, Inger Marie Jensen Hansen

**Affiliations:** a Department of Rheumatology, Odense University Hospital, Svendborg Hospital, Svendborg; b Faculty of Health Sciences, University of Southern Denmark; c Department of Nuclear Medicine, Odense University Hospital, Odense; d Department of Radiology and Nuclear Medicine, Hospital Southwest Jutland, Esbjerg; e Centre of Health Economics Research, University of Southern Denmark; f Department of Endocrinology; g Department of Rheumatology, Odense University Hospital, Odense; h Diagnostic center, Odense University Hospital, Svendborg Hospital, Svendborg; i Department of Pathology, Odense University Hospital, Odense; j Department of Ear Nose Throat Surgery; k Department of Radiology, Odense University Hospital, Svendborg Hospital, Svendborg; l Department of Rheumatology, Hospital Southwest Jutland, Esbjerg; m Patient Research Partner, Department of Rheumatology, Odense University Hospital, Svendborg Hospital, Svendborg, Denmark.

**Keywords:** fluorodeoxyglucose, giant cell arteritis, osteoporosis and malignancy, polymyalgia rheumatica, positron emission tomography/computed tomography, temporal artery biopsy, vascular stiffness, vasculitis

## Abstract

**Introduction:**

Polymyalgia rheumatica (PMR) and giant cell arteritis (GCA) are common inflammatory conditions. The diagnosis of PMR/GCA poses many challenges since there are no specific diagnostic tests. Recent literature emphasizes the ability of 18F-fluorodeoxyglucose positron emission tomography/computed tomography (18F-FDG PET/CT) to assess global disease activity in inflammatory diseases. 18F-FDG PET/CT may lead to the diagnosis at an earlier stage than conventional imaging and may also assess response to therapy. With respect to the management of PMR/GCA, there are 3 significant areas of concern as follows: vasculitis process/vascular stiffness, malignancy, and osteoporosis.

**Methods and analysis:**

All patients with suspected PMR/GCR referred to the Rheumatology section of Medicine Department at Svendborg Hospital, Denmark. The 4 separate studies in the current protocol focus on: the association of clinical picture of PMR/GCA with PET findings; the validity of 18F-FDG PET/CT scan for diagnosis of PMR/GCA compared with temporal artery biopsy; the prevalence of newly diagnosed malignancies in patients with PMR/GCA, or PMR-like syndrome, with the focus on diagnostic accuracy of 18F-FDG PET/CT scan compared with conventional workup (ie, chest X-ray/abdominal ultrasound); and the impact of disease process, and also steroid treatment on bone mineral density, body composition, and vasculitis/vascular stiffness in PMR/GCA patients.

**Ethics and dissemination:**

The study has been approved by the Regional Ethics Committee of the Region of Southern Denmark (identification number: S-20160098) and Danish Data Protection Agency (J.nr 16/40522). Results of the study will be disseminated via publications in peer-reviewed journals, and presentation at national and international conferences.

## 1 Introduction

Polymyalgia rheumatica (PMR) is the most common inflammatory rheumatic disease that affects individuals over the age of 50 years. The typical symptoms of PMR are bilateral aching and stiffness in selected muscle groups, predominantly in the neck, shoulders, upper arms, and pelvic girdle that are most pronounced in the morning and improve with physical activity.^[1–3]^ Giant cell arteritis (GCA) is an inflammatory vasculopathy that typically involves medium and large arteries.^[4]^ Though involvement of the extracranial branches of the carotid artery can be identified in the classic cranial GCA, GCA may also involve aorta and its major branches, predominantly branches of the proximal aorta, that is, subclavian, axillary proximal brachial arteries, representing a subset of patients with large-vessel GCA (LV-GCA).^[4,5]^ Vasculitis, if untreated, can result in ischemic complications, such as ischemic optic neuropathy, which is responsible for vision loss in 10% to 15% of patients. Whereas the etiology of PMR/GCA is still unknown, genetic and environmental factors may play roles in the development of PMR/GCA. GCA and PMR share many pathogenetic and epidemiological features, and are frequently thought to represent different spectrums of the same clinical entity. Despite the high association between PMR and GCA, it is essential to differentiate these 2 conditions due to different steroid treatment regimes. Corticosteroids are considered the treatment of choice for PMR/GCA, with higher doses required for GCA.^[1–3]^ PMR/GCA is generally not associated with reduced longevity; however, patients with aortic aneurysm/dissection have higher rates of mortality.^[6]^ Additionally, patients are at risk of different comorbidities, both due to the disease characteristic itself and also due to treatment side effects.

Accurate diagnosis of PMR/GCA can be challenging since various diseases mimic these conditions and there are no specific diagnostic tests. PMR is a clinical diagnosis. Careful clinical evaluation and exclusion of several mimics of PMR/GCA can support the diagnosis.^[1–3]^ In cases of suspected GCA, temporal artery biopsy (TAB) should be performed to confirm the diagnosis. However, it is invasive, it can be false-negative in a proportion of patients, and it does not delineate the extracranial component of the disease.^[7,8]^ Laboratory findings are nonspecific in PMR/GCA. Marked elevations in erythrocyte sedimentation rate (ESR) and C-reactive protein (CRP) are frequently seen in PMR/GCA. Thrombocytosis, anemia, and increased alkaline phosphatase may also be presented.^[1,3]^ Different imaging modalities, for example, ultrasonography (US), magnetic resonance imaging, and angiography, are helpful in the diagnosis of PMR/GCA; however, use of these modalities may be limited due to long assessment time for wide vascular assessment, interobserver variation, invasiveness, diminished sensitivity, specificity, and so on.^[9–11]^ There have been several proposed diagnostic criteria for PMR.^[12–15]^ The core components included in these diagnostic criteria were as follows: an age cut-off point, bilateral shoulder or hip pain, morning stiffness, elevated ESR, elevated CRP, rapid steroid response, and disease duration. In 2012, the provisional classification criteria for PMR has been published by European League Against Rheumatism (EULAR) and American College of Rheumatology (ACR).^[10]^ However, it is worth mentioning that these are classification criteria and are designed to differentiate PMR from other diseases that mimic PMR and are not useful to make the diagnosis. Furthermore, the specificity of the 2012 provisional classification criteria for PMR seems to be not sufficient, despite high sensitivity.^[16]^

Positron emission tomography/computed tomography (PET/CT) is a whole-body scan combining molecular and structural imaging in a single examination. The principle is tomographic detection of radiation from intravenously administered radio-labeled tracers targeting disease processes on a cellular or molecular level. Due to its high sensitivity and an inborn ability for quantification, PET can detect diseases at their very early stage and grade their degree of activity.^[17,18]^ The most versatile and widely used tracer, 18F-fluorodeoxyglucose (FDG), is a glucose analog depicting local hypermetabolism (ie, inflammation or cancer). In the clinic, it has mainly been used to detect focal hypermetabolism due to cancer, and the impact in the oncologic domain has been huge as PET/CT appears to cause a change in management in as many as every third patient.^[19]^ However, FDG is nonspecific for cancer as inflammatory lesions also show increased FDG uptake. This was until recently seen as a downside resulting in “false-positive” findings in oncology, but is now considered a great asset, since this allows for detection and characterization of inflammatory processes in many settings. 18F-FDG PET/CT is being investigated clinically in a multitude of inflammatory disorders. High sensitivity, high target-to-background contrast, accurate anatomical localization of sites of inflammation, and an absolute quantification potential may all lead to early assessment of disease processes and their extent.^[20]^

With regard to vasculitis, recent literature emphasizes the ability of 18F-FDG PET/CT to assess inflammation in the vessel walls, whereas FDG uptake patterns in PMR are not yet firmly established. It may locate disease at an earlier stage than conventional imaging and assess response to therapy. Response assessment is based on changes in glucose metabolism from baseline to post-treatment follow-up, and the rates of change are different in responders and nonresponders.^[21]^ Besides locating focal disease, 18F-FDG PET/CT may also assess disease activity more globally, which may be especially interesting in systemic inflammatory diseases. Thus, this research protocol will also incorporate assessment of global disease assessment (ie, “global disease burden”), a novel approach to quantifying disease. By way of novel software-based quantitative methods, the radioactivity throughout the body can be quantified, summed, and presented as 1 single number, the systemic inflammatory activity or “global disease burden.” The method is new and not yet firmly established, but it holds significant promise, especially for response assessment in systemic inflammatory diseases.^[22,23]^

**Figure 1:**
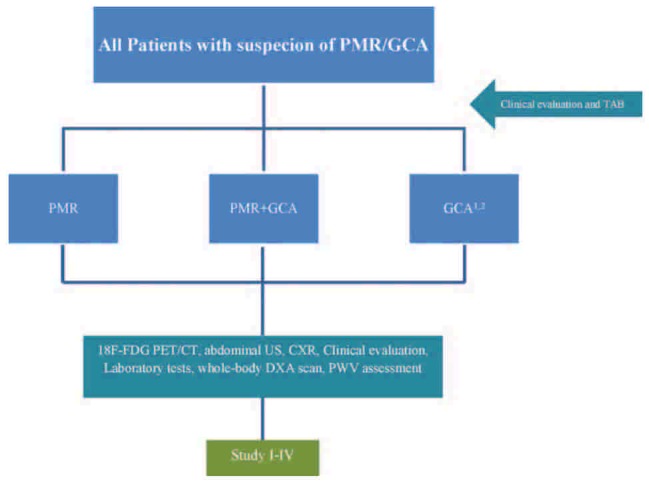
Summary of study I to IV. GCA = giant cell arteritis, PMR = polymyalgia rheumatica, TAB = temporal artery biopsy.

Therefore, we plan to conduct a prospective study to explore the role of 18F-FDG PET/CT scan in the management of PMR/GCA. In addition, 3 significant areas of concern in the management of PMR/GCA including: osteoporosis due to steroid treatment or disease characteristics itself, malignancy, and vasculitis/vascular stiffness will be investigated during the project. This research protocol consists of four separate studies designed to achieve below mentioned objectives that are discussed individually later in the text (Fig. 1).

## 2 Objectives

### 2.1 Primary

The primary objective of this study is to explore the association of 18F-FDG PET/CT findings and uptake patterns (PET positivity/negativity) and the clinical picture of PMR/GCA.

### 2.2 Secondary

1. To investigate the validity of 18F-FDG PET/CT scan for diagnosis of PMR/GCA compared with temporal artery biopsy.
2. To compare the efficacy of nationally recommended conventional diagnostic workup to detect occult malignancies in the setting of PMR/GCA (ie, chest X-ray [CXR] plus abdominal US) with 18F-FDG PET/CT at diagnosis and during the first year after diagnosis.
3. To evaluate the influence of inflammation on regional and total bone mineral density (BMD) and body composition, and also vasculitis/arterial stiffness of aorta at diagnosis of PMR and within a year after treatment initiation with prednisolone.

### 2.3 Patient research partners (PRPs)

Active collaboration between patients and researchers is relatively a new concept in the research field which is a useful tool to capture the patient perspective. By means of this, better representation of patients’ needs and uncertainties can be secured. It additionally avoids potential mismatch between patients’ preferences and researchers objectives. EULAR has introduced the recommendation for the inclusion of patient representatives in scientific projects.^[24]^ This research protocol will also adhere to the EULAR recommendation. Thus, 2 PRPs (co-authors Hanne Andersen and Rudolf Olsen) were invited to help us design the study. The involvement of PRPs with respect to 8 significant aspects of EULAR recommendation is depicted in Table 1.

**Table 1:**
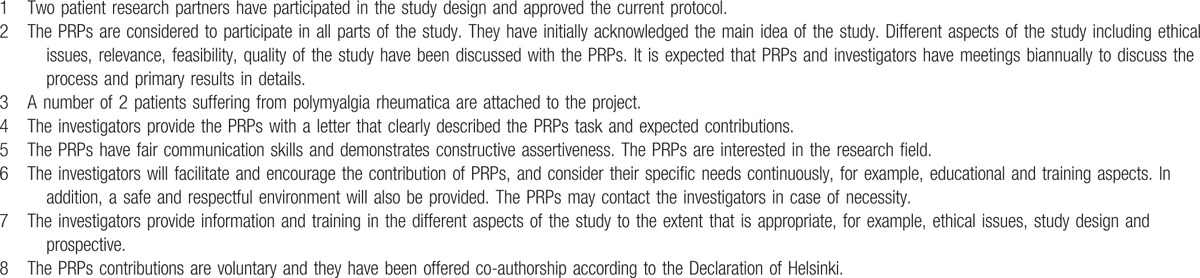
Patient research partners (PRPs).

## 3 Methods and analysis

### 3.1 Study setting

The current study will be performed at the Department of Rheumatology, Svendborg Hospital, Odense University Hospital, Svendborg, Denmark. The Rheumatology Department of Svendborg Hospital has the capacity of enrolling PMR/GCA patients that are required for this study, since all patients from all over Funen County with suspicious PMR/GCA will be referred to the Diagnostic Center for primary investigation at Svendborg Hospital. After confirmed PMR/GCA condition the patients will be referred to the local Rheumatology department for study inclusion.

All parts of the project will take place at Svendborg Hospital, except the 18F-FDG PET/CT scans and bone densitometry, which will be undertaken at the Department of Nuclear Medicine and Department of Endocrinology, respectively, Odense University Hospital, Odense, Denmark. The 18F-FDG PET/CT will be performed as routine whole-body scan (from vertex of the skull to the knees) 60 minutes after injection of a weight-adjusted dose of FDG according to the guidelines from the European Association of Nuclear Medicine.^[25]^ The CT-part is a low-dose CT without contrast enhancement.

### 3.2 Length of study (study I–IV)

The study was conducted till 1 year after the enrollment of last patient.

### 3.3 Participants

All new patients with clinical suspicion of PMR/GCA will be offered to be included in the study. At least 5 (A–E) components of the PMR diagnostic criteria, including: age ≥50 years, bilateral shoulder or hip pain, morning stiffness lasting >45 minutes, elevated ESR, elevated CRP, disease duration >2 weeks, should be met to suspect PMR.^[3]^ For GCA, the following criteria must be considered: age >50 years, ESR/CRP >50, and also at least 2 symptoms related to vasculitis (scalp tenderness, vision disturbances, headache [new or changed], jaw claudication, tenderness of the temporal arteria) if patients do not simultaneously have PMR. If the patient is suspected for PMR, 1 cranial symptom is enough to suspect GCA. Besides, clinical suspicion of LV-GCA, that is, upper-extremity claudication, upper-extremity blood pressure discrepancies, and abnormal radial pulse, will be registered.

The exclusion criteria (study I–IV) are as follows:

1. Patients with dementia or inability to communicate in Danish
2. Infections or malignancy when prednisolone is permanently unsuitable
3. Contraindication to imaging studies (pregnancy and blood sugar [BS] >8 mmol/L after 6 hours fasting)
4. Initiation of steroid treatment before the 18F-FDG PET/CT scan
5. Inability to provide informed consent

### 3.4 Study design

#### 3.4.1 Study I: the association of clinical picture of polymyalgia rheumatica and giant cell arteritis with 18F-FDG PET/CT findings: 1-year, prospective, blinded explorative study

The current study is a 1-year prospective, blinded, and explorative study. This is the first study of its kind, to our knowledge, to evaluate the association between the clinical picture of PMR/GCA and 18F-FDG PET/CT findings in a prospective setting. Previous study revealed the correlation between serological markers of inflammation and FDG accumulation in PMR.^[7,26–28]^ However, the small sample size was a major limitation. On the contrary, some reports failed to show this correlation.^[9,29,30]^

All included patients initially will undergo an 18F-FDG PET/CT scan. The results of the 18F-FDG PET/CT scans will not be made accessible to the treating physician in daily clinical routine, but unexpected findings or findings requiring further diagnostic tests or intervention will be relayed to the chief physicians responsible for inclusion. The results of 18F-FDG PET/CT scan will be blinded for both the clinicians and patients. During the year after the initial 18F-FDG PET/CT scan, the following clinical data will be monitored: cumulated prednisolone doses (using patient diaries), well-being of the patients (including visual analog scale [VAS], patient's assessment of pain, morning stiffness [minute], VAS for physicians’ evaluation), ESR, CRP, fibrinogen, and the eventual number of relapses. Definition of remission and relapse is based on expert opinion considering the following parameters: patient's assessment of pain, morning stiffness, ESR, CRP, clinical examination findings (eg, shoulder pain/limitation, upper extremity blood pressure discrepancies, vascular bruits and aortic regurgitation murmur), and steroid dose which is required to control symptoms.

After 1 year, the 18F-FDG PET/CT results will be unveiled and these parameters will be compared in the succeeding groups of patients: patients with vasculitis in the large vessels (PET-positive); patients without vasculitis in the large vessels (PET-negative); patients with findings compatible with PMR (PET-positive); and patients with vasculitis in the large vessels together with findings compatible with PMR (PET-positive). The classification of 18F-FDG PET/CT results (PET +/−) is based on visual assessment in a descriptive manner, and also quantitative methods. The visual assessment is based on simple visual assessment of FDG uptake patterns in a descriptive manner (PMR) and a 4-point scale of FDG uptake in the vessels (GCA). The visual assessment will be compared with simple semiquantitative analyses, that is, so-called maximum standard uptake value (SUVmax), a simple delineation of the single voxels with the highest tracer uptake. This is a simple procedure available with the generic software indigenous to the scanners and provided by the vendors, for example, in our case, GE Advantage Server 2.0 (GE Healthcare, Milwaukee, WI). It is easily applicable in clinic, but highly variable and prone to both technical and patient related pitfalls. Thus, a third method of quantification will be employed and compared with the visual assessment and SUVmax, that is, global disease burden,^[31]^ which is based on more elaborate, and therefore technically cumbersome, computations involving segmentation, quantification, and summing of all FDG-avid lesions with pathologic activity defined by predetermined threshold. These metabolically active volumes (MAVs) will be summed to yield a global disease score, a metabolic volume product (MVP) of all PET-positive lesions throughout the body. This seems to be a very robust method, but also somewhat cumbersome to implement in daily clinical practice.

##### 3.4.1.1 Image analysis

Images will be reviewed by 2 nuclear medicine specialists at the Department of Nuclear Medicine at Odense University Hospital who are blinded to clinical and laboratory data. FDG uptake patterns will be recorded in a descriptive manner with regard to nonarterial uptake and recorded in 6 arterial compartments for each individual patient including thoracic and abdominal aorta, carotid, subclavian, femoral, and iliac arteries. A visual 4-point scale, as described by earlier studies,^[7,27–29]^ will be used for every arterial segment as follows:

0: No uptake
1: Slight uptake but not negligible, lower than liver uptake
2: Intermediate uptake, similar to liver uptake
3: High-grade intense uptake, higher than liver uptake

A score of ≥2 and <2 will be considered PET-positive and PET-negative, respectively. SUVs will be measured for quantitative analysis for each arterial segment and calculated as follows: 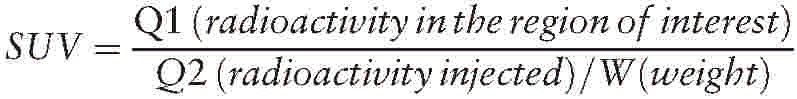


Global disease burden will be expressed as follows:

cMVP = ∑ (MAV∗cSUVmean), where

cMVP = partial volume corrected metabolic volume product,

MAV = metabolic active volumes, and

cSUVmean = partial volume corrected mean standard uptake value

#### 3.4.2 Study II: evaluating validity of 18F-FDG PET/CT scan for diagnosis of polymyalgia rheumatica and giant cell arteritis compared to temporal artery biopsy: an agreement study

Recent literature is in favor of the ability of 18F-FDG PET/CT scan to detect vasculitis at an early stage, which is important to decide on treatment plan.^[32]^ However, the superiority of 18F-FDG PET/CT scan in comparison with TAB is still arguable. This study will compare 18F-FDG PET/CT scan to TAB. As mentioned in the study I, all included patients will undergo an 18F-FDG PET/CT scan. In addition to 18F-FDG PET/CT scan, TAB will be performed before or within 1 week of steroid treatment initiation. TAB will be taken by experienced otolaryngologist at Department of Ear, Nose and Throat, Svendborg Hospital. The results of biopsy will be compared with the 18F-FDG PET/CT findings after 1 year, when the 18F-FDG PET/CT results are revealed and compared with regards to the degree of agreement between the 2 modalities.^[33,34]^

##### 3.4.2.1 Temporal artery biopsy procedure

In patients with lateralizing symptoms, the biopsy will be taken from the symptomatic side. The superficial temporal artery will be palpated and marked with a surgical marker. The site will be shaved if is necessary. Local anesthetic will be injected into the skin and subcutaneous tissue corresponding to planned incision using lidocaine 1% with or without adrenalin. Under sterile condition the incision will be made at the previously marked skin site. The incision should be large enough to uncover 2 to 3 cm of the vessel. The dissection will be carried through the subcutaneous fat and the artery will be identified just beneath the temporoparietal fascia. The artery will be ligated proximally and distally, and so the intervening segment will be cut out and fixated in formaldehyde. The in vivo specimen should be minimum 1.5 cm in length. At last, skin will be sutured using non-absorbable suture material.

##### 3.4.2.2 Preparation of specimens for pathologic examination

The biopsy will be fixed in formalin for 24 hours, divided in sections of 3 to 4 mm, embedded in paraffin, cut in step sections, and stained with Weigert elastin and hematoxylin and eosin (H&E), respectively. Additional sections will be cut for later analysis. Some of the additional sections will include deeper sections in the block to further evaluate the extent of the lesion. The additional analysis will include immunohistochemistry for inflammatory cells, proliferation markers and markers of fibrosis.

#### 3.4.3 Study III: prevalence of newly diagnosed malignancies in patients with polymyalgia rheumatica and giant cell arteritis, or polymyalgia rheumatica like syndrome with a focus on 18F-FDG PET/CT compared with CXR/abdominal US: a blinded, prospective, explorative study

The relationship between PMR/GCA and malignancies remains controversial. There are several reports regarding the association of PMR and malignancies. In a database study from Sweden, where 35,918 patients after hospitalization for GCA and PMR were included, there was a marginally increased incidence of cancer in patients with GCA compared with the general population (standardized incidence ratio 1.19, 95% confidence interval [CI] 1.06–1.23). However, when evaluating malignancy risk in the first year after diagnosis of PMR or GCA, the standardized incidence ratio was 2.26 (95% CI 2.10–2.42).^[35]^ Another study utilized the UK General Practice Research Database and evaluated cancer risk in 2877 patients with PMR, without pre-existing cancer or vascular disease and treated with corticosteroids, who were matched with up to 5 age, sex, and general practice patients without PMR. Over a median follow-up of 7.8 years, 23.2% of patients with PMR developed cancer compared with 19.5% of controls. The risk of malignancy was increased in patients with PMR during the first 6 months after diagnosis (hazard ratio [HR] 1.69, 95% CI 1.18–2.42).^[36]^ However, the risk of misclassification/misdiagnosis in database studies is notable, which also points out the need for studies with prospective design. Danish Rheumatology Association recommends the screening for malignant diseases in PMR/GCA patients.^[3]^ Various malignancies can cause PMR-like symptoms; therefore detailed assessment to exclude occult malignancies seems to be rational, especially in patients with atypical manifestations.^[37,38]^ Patients with malignant disease may also present with remitting seronegative symmetrical synovitis with pitting edema, which is a closely related condition to PMR.^[39,40]^

This study is a blinded prospective investigation. At diagnosis all patients undergo a CXR and abdominal US, as recommended by the Danish Rheumatology Association. The 18F-FDG PET/CT scan in every included patient (as described in study I) will be assessed with regards to malignancies before results are blinded to the clinicians for 1 year. This assessment will be blinded to other clinical and imaging results and findings on 18F-FDG PET/CT scan will be compared with CXR/abdominal US results to establish the relative diagnostic accuracy of these 2 strategies for malignant and infectious diseases in PMR/GCA patients. In case of detecting or developing any kinds of malignant disease during the study, the patients will be referred to the relevant department for further work-up and treatment. This will not be a reason for exclusion, but depends on individual assessment of the patient. 18F-FDG PET/CT scan may also be repeated for individual patient if necessary.

#### 3.4.4 Study IV: consequences of steroid treatment for BMD, body composition, and also vasculitis/vascular stiffness in polymyalgia rheumatica and giant cell arteritis patients: 1-year prospective study

Patients who are diagnosed with PMR/GCA are at high risk of osteoporosis due to both disease inflammatory process and corticosteroid treatment. Nonspecific bone protection (lifestyle intervention) should be initiated at the time of steroid therapy, and specific antiosteoporotic therapy soon thereafter in patients with low BMD. The gold standard for the diagnosis of osteoporosis is regional dual energy X-ray absorptiometry (DXA) of the hip and spine. Whole-body DXA can estimate 3 body compartments consisting of fat mass (FM), lean body mass (LBM), and bone mass. DXA is currently considered the gold standard for body composition assessment. Changes in whole body bone mineral content (BMC) is a biological meaningful measure of overall calcium balance over a time period, whereas changes in regional BMD are probably more predictive for fracture risk. Changes in body composition due to inflammatory process have previously been reported in rheumatoid arthritis.^[41–43]^ Patients with PMR/GCA are also exposed to different changes in body compositions mainly due to inflammatory process; however, this has not been evaluated before.

The vasculitis process in patients with classic cranial GCA preferentially involves the extracranial branches of the carotid artery; however, large vessel involvement of the aorta and its primary and secondary branches may be involved in patients with LV-GCA.^[4]^ The extent of large artery involvement of GCA is uncertain, but clinically apparent large-vessel disease comprises an estimated 9% to 14% of cases. ^[44,45]^ LV-GCA patients have symptoms and signs of vascular insufficiency and are usually diagnosed with delays due to atypical manifestations, and also inadequacy of GCA criteria for classifying these patients.^[4]^

Arterial distensibility has been defined as a measure of the artery's ability to expand and contract with cardiac pulsation and relaxation. Vasculitis and other risk factors (eg, aging, hypertension, and arteriosclerosis) may cause alteration in the structural and functional properties of the arterial wall, which results in diminished arterial distensibility. When evaluating arterial stiffness, the aorta is a main vessel of interest, because of substantial contribution of abdominal and thoracic aorta to the arterial buffering function.^[46,47]^ Pulse wave velocity (PWV) measurement is a simple, noninvasive, and reproducible method to evaluate aortic stiffness, and can be measured from different arterial sites. Common carotid artery and the femoral artery are 2 common sites to obtain pressure waveforms transcutaneously. The distance covered by the waves is corrected to the surface distance between the 2 recording sites and base of aorta (eg, jugulum), and the time delay (or transit time) is measured using the feet of the 2 waveforms (at the end of diastole, when the steep rise of the wave front begins). PWV is subsequently measured as the ratio of the distance to the time delay (PWV [m/s] = distance [m]/transit time [seconds]).^[46]^ Previous study was in favor of that PMR is associated with increased aortic stiffness which may improve upon reduction of systemic inflammation induced by treatment with glucocorticoids.^[48]^

This study is a 1-year prospective investigation which assesses 2 different areas of concern in PMR/GCA patients, namely BMD plus body composition and vasculitis/vascular stiffness of large vessels (aorta). The following procedures will be performed in all included patients:

1. PWV measurement of aorta at baseline and then every 3 months to assess large vessels involvement (aorta). The results of PWV assessment, which will be automatically calculated from measurements of pulse transit time and distance between the 2 sites, will be compared with vasculitis found in the 18F-FDG PET/CT (study I). The results of PWV of aorta will also be assessed regarding the actual cumulated prednisolone dose to find out the effects of steroid treatment on the arterial stiffness.
2. Whole-body DXA scan at baseline and after 1 year from the initiation of study to evaluate BMD and body compositions. The degree of the difference in the T score and body composition (including lean body mass, fat mass, bone mineral content, and BMD) will be compared with the cumulated prednisolone dose and the total global disease burden of the 18F-FDG PET/CT scan at baseline (study I).

##### 3.4.4.1 Body composition measurement

Body mass index (BMI) of patients will be calculated from weight/height^2^. Individuals with BMI values <18.5 kg/m^2^ are considered underweight, 18.5 ≤ BMI < 25 as normal, 25≤ to <30 as overweight. BMI values ≥30 indicates obesity.^[49]^ LBM, FM, BMD, BMC, and fat-free mass (FFM = LBM + BMC). FM and FFM will be expressed as absolute kg and FM as percentage of total mass as well. The reference value for FM% is 12% to 20% for men and 20% to 30% for women.^[50]^ FFM and fat mass indices (FFMI and FMI) will also be calculated. An aged and sexed matched European reference from Switzerland will be used for cut-off values for being under lean and obese.^[51]^

Bone mineral density values will be presented as Z scores (the number of standard deviations from the mean in healthy age and sex-matched people) and T scores (the number of standard deviations from the mean in healthy young sex-matched people). T score ≤−2.5 will be considered as osteoporosis and T score <−1 as osteopenia.

##### 3.4.4.2 PWV assessment

After measuring blood pressure (BP), aortic (aorta to femoral) and upper limb (aorta to radial) PWV will be measured using SphygmoCor device. Aortic PWV will be calculated from measurements of bulbus aorta and femoral artery, and upper limb PWV will be calculated from measurements of bulbus aorta and radial artery waveform. Aortic augmentation index (the difference between the second and first peaks of the central arterial waveform expressed as a percentage of the pulse pressure) with a standardization to a heart rate of 75 beats per minute will also be assessed, which is a predictor of adverse cardiovascular events.

### 3.5 Clinical evaluation and laboratory tests (study I–IV)

Blood pressure, pulse, weight, and also BMI of all patients will be collected at the first visit. The following laboratory tests will be recorded: hemoglobin, white blood cells and thrombocytes, erythrocyte sedimentation rate, C-reactive protein, sodium, potassium, lipid profile, thyroid-stimulating hormone (TSH), hemoglobin A1c (HbA1C), blood sugar, creatinine, liver function test (LFT), alkaline phosphatase, calcium, vitamin D, creatine kinase, immunoglobulin A, immunoglobulin M, immunoglobulin G, antinuclear antibody (ANA), antineutrophil cytoplasmic antibody (ANCA), IgM rheumatoid factor (IgM RF), anticyclic citrullinated peptide (anti-CCP), S-fibrinogen, prostate-specific antigen (PSA), M component (blood and urine), serum-free light chains (sFLC), urine protein, urine analysis, and electrocardiogram.

### 3.6 Treatment plan

#### 3.6.1 Steroid treatment

Steroid treatment is based on a modification of the Danish Rheumatology Association Guidelines. Dose reduction or dose increase is also according to this guideline. Prednisolone will be initiated after performing 18F-FDG PET/CT scan (Table 2).

**Table 2:**
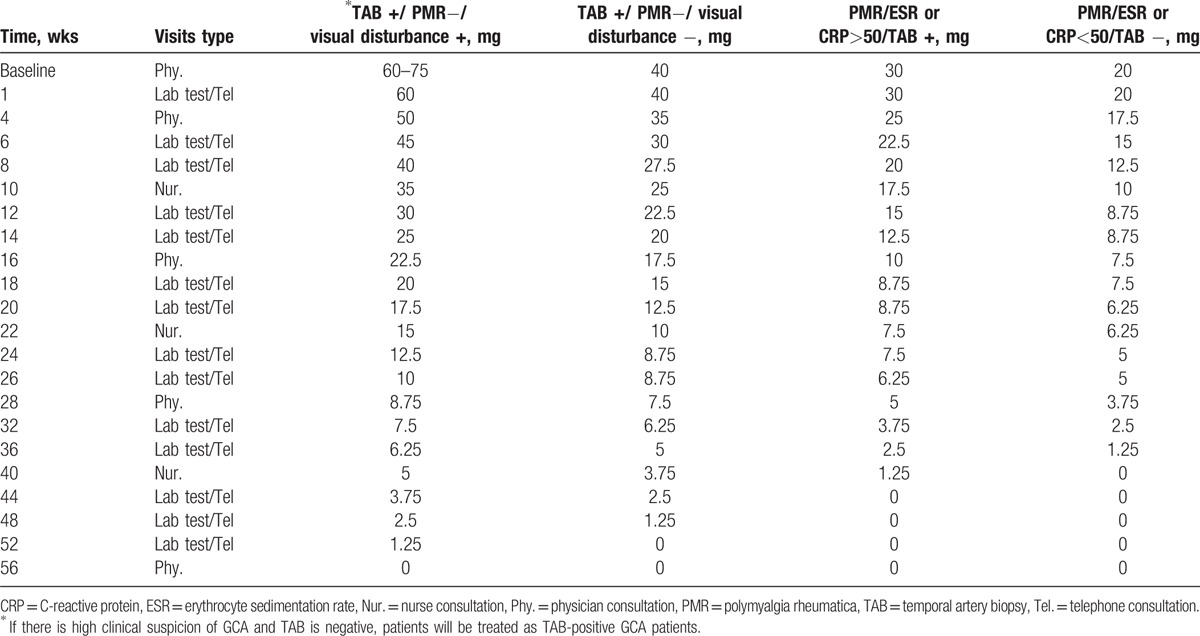
Steroid treatment and types of visits for the included patients (in case of persistent response, normal CRP and no clinical symptoms).

#### 3.6.2 Osteoporosis prevention

Calcium (1200 mg/d) plus vitamin D (800 U/d) and 70 mg alendronate weekly (if Tscore <−1) will be prescribed to reduce risk of fracture.

### 3.7 Follow-up (study I–IV)

Detailed follow-up plan is summarized in Table 3.

**Table 3:**
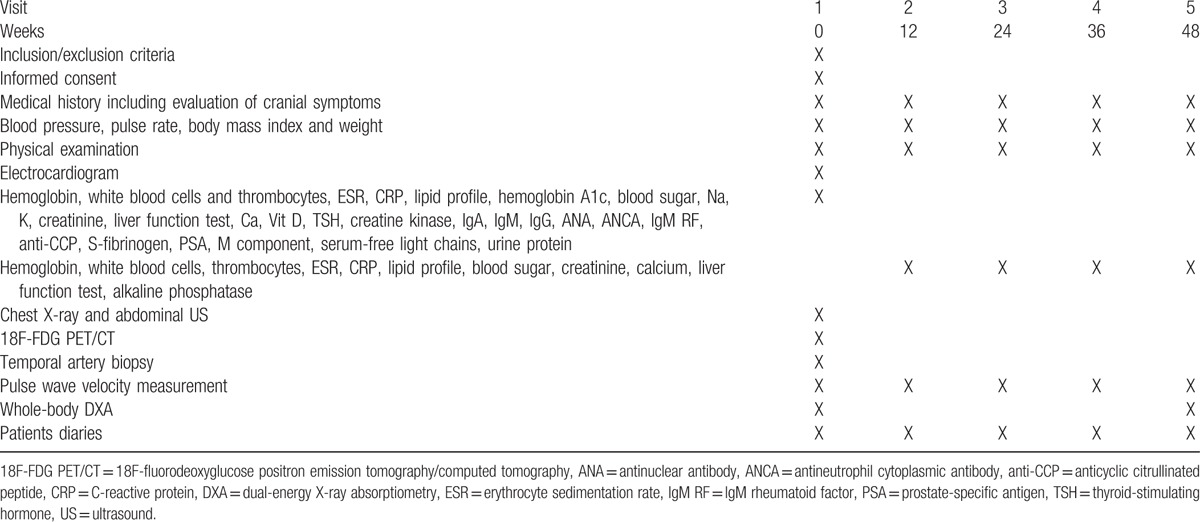
Schedule of assessment during the study.

In addition to the mentioned investigations, patients will be invited in a coming investigation to perform a magnetic resonance of aortae, and also heart CT scan after completion of the study, to evaluate the risk of aneurisms, especially in the patients with PET positivity of the large vessels. Furthermore, a PWV measurement will also be done at that time.

### 3.8 Outcomes

The primary outcome measure are as follows:

1. To evaluate the cumulated prednisolone doses, VAS, patients’ assessment of pain, morning stiffness, VAS for physician evaluation, biochemistry results, that is, ESR, CRP, and fibrinogen, and the eventual number of relapses at baseline and during the first year after treatment initiation (weeks 12, 24, 36, and 48) in patients with PET-positive and PET-negative as described above.

The secondary outcome measures are as follows:

1. To elucidate the degree of agreement between PET positivity (vasculitis in the large vessels, and also findings compatible with PMR) and TAB positivity.
2. To elucidate the degree of agreement between PET negativity (no signs of vasculitis in the large vessels) and TAB negativity.
3. To reveal the prevalence of malignancies in the patients detected by the aim of 18F-FDG PET/CT scan and subsequently compare to the prevalence of malignancies detected by the aim of CXR plus abdominal US.
4. To reveal the prevalence of malignancies in the patients detected by the aim of CXR plus abdominal US and subsequently compare to the prevalence of malignancies detected by the aim of 18F-FDG PET/CT scan.
5. To evaluate BMI, T score, Z score, LBM, FM, BMD, BMC, FFM, FFMI, and FMI at baseline and after 1 year of treatment initiation.
6. To evaluate aortic PWV, upper limb PWV, and aortic augmentation index of included patients at baseline and during treatment (weeks 12, 24, 36, and 48).

### 3.9 Sample size

This study is, to the best of our knowledge, the first of its kind; hence, formal power calculations were impossible to conduct due to missing information from previous literature or local clinical daily routine. In this exploratory study, the number of patients to be included was set to 80, which enables the assessment of the primary objective, that is, the difference in the cumulated prednisolone dose within the first year after diagnosis between groups including a respective 95% CI, with sufficient precision. Moreover, 80 patients are deemed appropriate to assess the secondary objectives for hypothesis generating purposes.

### 3.10 Recruitment

All patients with the suspicion of PMR/GCR are referred to the Diagnostic Center, Svendborg Hospital. After primary investigation and evaluation, the eligible patients will be referred to the Rheumatology Department of Svendborg Hospital for formal enrolment in the study. Patients will be informed orally and written about the voluntary nature of the participation and project objectives, content, risks, and benefits. A deliberation time of 24 hours will be considered from submission of the oral and written information to obtain informed consent. Consent statement will be reviewed, signed, and dated by the patient and handed over to the department. The patient receives a signed copy of the consent form and be informed of that he/she can withdraw from the study at any time without any reason nor consequences. A scanned copy of patient consent will be added to the patient electronic hospital record, and one additional copy will be stored in a locked room at the department.

### 3.11 Data collection

A PhD student will be responsible for collecting data elements from patient medical records, including: demographic data, vital signs (BP, PR), weight, BMI, results of laboratory tests, ECG, and diagnostic tests (18F-FDG PET/CT scan, CXR, abdominal US, TAB, PWV assessment, and whole-body DXA scan), and also cumulative prednisolone dose. Data will be entered into the University of Southern Denmark Research Electronic Data Capture (REDcap) database twice to minimize data entry errors.

### 3.12 Statistical analysis

It is anticipated that 80 participants are likely to be included during a period of 1 year. The results will be considered as significant if *P* value <0.05. Statistical analysis has been discussed below with respect to each individual study.

In study I, data will be descriptive (mean ± standard deviation). Comparisons of the data, between the PET-positive and PET-negative patients, will be made with the Mann–Whitney *U* test or Student *t* test, depending on whether or not the distribution is normal. When comparing 2 binary variables, the chi-square test will be performed. Correlation analysis will be performed with Pearson correlation test.

Study II consists of descriptive statistics and proportions of agreement.

Study III consists of descriptive statistics. Comparisons between groups will be made with McNemar test. The sample size of 80 patients is sufficient to indicate a difference of 15% in detection rate of malignancies with 18F-FDG PET-CT scan as opposed to CXR plus abdominal UL (with a significance level of 5% (1-sided), power of 80%, and assuming discordant proportions of 15% and 0%).^[52]^

Study IV consists of descriptive statistics. Univariate comparisons between groups will be made with the Mann–Whitney *U* test or Student *t* test, depending on whether or not the distribution is normal. When comparing 2 binary variables, the chi-square test will be performed. Analysis of repeated measurements over time will be done by means of mixed linear models, using standard errors that allow for intragroup correlation, relaxing the usual requirement that the observations be independent. Comparison of the above mentioned baseline data, matched by age and sex, will be made, to the extent that these data are available.

### 3.13 Ethical consideration

Initial and control blood samples will be taken regarding the routine management of PMR/GCA patients. There is no further risk, for example, pain or infection for included patients compared with other patients not participating in this study. The outpatient visit may take longer time due to systematic history taking and physical examination, PWV assessment, and so on, which is a disadvantage for a busy patient.

An 18F-FDG PET/CT scan exposes a patient to about 8 mSv radiation. The average per capita background radiation is 4 mSv per year in Denmark. The average lifetime risk of cancer is approximately 25% for a 25-year-old person in Denmark. The radiation dose from one 18F-FDG PET/CT scan increases this risk to approximately 25.01%. In persons above 50 years of age, the increased risk is even less.

Due to increased rate of malignancy in PMR/GCA, patients may benefit from 18F-FDG PET/CT scan, which detects possible concurrent malignancies.

Whole-body DXA scan will be performed at baseline and a year after which is appropriate for patients receiving steroids. Evaluating fat mass takes place during the same session and extends the study by about 5 minutes which is acceptable. The radiation dose of whole-body DXA is negligible.

Vascular stiffness will be measured by SphygmoCor in conjunction with routine outpatient controls. This method is completely noninvasive and takes about 15 to 30 minutes.

Although performing 18F-FDG PET/CT scan together with TAB is a disadvantage of this study, patients are hopefully not required to do TAB in future. TAB may still be required in small number of patients.

### 3.14 Research ethics approval

The present study protocol is approved by the Regional Ethics committee of the Region of Southern Denmark (identification number: S-20160098) and Danish Data Protection Agency (J.nr 16/40522). This study is also registered at clinicaltrials.gov (ClinicalTrials.gov Identifier: NCT02985424).

### 3.15 Protocol amendment

Any modifications to the protocol which have substantial effects on the conduct of the study, potential benefits to the patient or impact on patient safety, including changes to eligibility criteria, patient population, sample size, study objectives, study design, study procedures, and treatment plan will require a formal amendment to the protocol. Such amendment will be sent to the Ethics committee for further approval before implementation. Administrative changes of the protocol defined as minor corrections having no effect on the way the study is to be conducted will be documented and the Ethics committee may also be notified of them.

### 3.16 Consent

The PhD student is responsible for obtaining informed consent from potential participants or authorized surrogates. All participants will be informed about the voluntary nature of the participation in the study and will be received detailed information about diagnostic and treatment modalities.

### 3.17 Confidentiality

The PhD student will give a unique code to each participant which will be used to identify them during the study. Any kinds of paper reports, laboratory specimens, administrative form, and so on will be identified by theses codes. All data collection sheets, patients consent forms, daily registration chart, and so on will be secured in an office with limited access. All digital files will be saved in our local databases which are password protected.

### 3.18 Dissemination

The current study is a large-scale study and will form the basis of a PhD thesis. The hard copy of our work will be available at the University of Southern Denmark library once completed. The results of the study are intended for the following potential papers (preliminary titles) that will be sought published in international peer reviewed journals, or presented at International or national congresses orally.

1. The clinical picture in PMR/GCA patients with and without PET detected vasculitis.
2. Validity of 18FDG PET-CT scan for diagnosis of PMR/GCA compared with TAB: An agreement study.
3. Prevalence of newly diagnosed malignancies in PMR/GCA patients with the aim of 18F-FDG PET/CT scan and CXR/Abdominal US: A blinded prospective study.
4. Effects of steroid treatment on Bone mineral density and body composition in PMR/GCA patients: A prospective study.
5. Effects of steroid treatment on vasculitis/vascular stiffness of PMR/GCA patients with the aim of PWV measurement: A prospective study.

After the completion of data collection, no later than 3 years, we will deliver a deidentified dataset to a suitable data archive for sharing purposes.

## 4 Discussion

The proposed study will explore the potential role of 18F-FDG PET/CT scan in the early diagnosis and management of PMR/GCA before initiation of steroid treatment, and also the association between 18F-FDG PET/CT findings and the clinical picture of the patients. Though 18F-FDG PET/CT is not yet capable of evaluating temporal artery due to inadequate resolution, the extracranial involvement of large vessels, mainly thoracic aorta, carotid, and subclavian, can be delineated.^[53]^ Patients with positive 18F-FDG PET/CT findings will be classified as having GCA and not isolated PMR, which is of great importance in the clinic due to different steroid treatment regimes. Besides, GCA patients with aortic involvement are at increased risk of aneurysm formation, who seek close monitoring.^[54]^ It is our hope that our results elucidate the 3 main areas of concern regarding the management of PMR/GCA including: malignancy, osteoporosis/changes in body composition, the vasculitis process, and the interaction between them, and ultimately elucidate factors predictive of the disease outcome.

## Acknowledgment

We thank Professor Kim Hørslev-Petersen, DMSci, for his insightful comments.
